# Supplementary material for: Resolving the full spectrum of human genome variation using Linked-Reads
Source: Genome Res. 2019 Apr;29(4):635–45. doi: 10.1101/gr.234443.118 (PMC6442396; doi:10.1101/gr.234443.118)
Supplement: Supplemental Material [file supp_gr.234443.118_Supplemental_longranger-2.2.zip › longranger-2.2.tar.bz2!/lr2.2-public/lib/stan/cmdstan/stan/doc/api/html/base__hamiltonian_8hpp_source.html]

Stan: src/stan/mcmc/hmc/hamiltonians/base\_hamiltonian.hpp Source File


|  |  |
| --- | --- |
|  | Stan  2.5.0  probability, sampling & optimization |


- Main Page
- Related Pages
- Namespaces
- Classes
- Files

- File List
- File Members

All Classes Namespaces Files Functions Variables Typedefs Enumerations Enumerator Friends Macros Pages

- src
- stan
- mcmc
- hmc
- hamiltonians

base\_hamiltonian.hpp

Go to the documentation of this file.

1 #ifndef STAN\_\_MCMC\_\_BASE\_\_HAMILTONIAN\_\_BETA

2 #define STAN\_\_MCMC\_\_BASE\_\_HAMILTONIAN\_\_BETA

3

4 #include <stdexcept>

5 #include <fstream>

6 #include <iostream>

7 #include <vector>

8

9 #include <stan/math/matrix/Eigen.hpp>

10 #include <stan/model/util.hpp>

11

12 namespace stan {

13

14  namespace mcmc {

15

16  template <typename M, typename P, typename BaseRNG>

17  class base\_hamiltonian {

18

19  public:

20

21  base\_hamiltonian(M& m, std::ostream\* e): model\_(m), err\_stream\_(e) {};

22  ~base\_hamiltonian() {};

23

24  virtual double T(P& z) = 0;

25  double V(P& z) { return z.V; }

26

27  virtual double tau(P& z) = 0;

28  virtual double phi(P& z) = 0;

29

30  double H(P& z) { return T(z) + V(z); }

31

32  // tau = 0.5 p\_{i} p\_{j} Lambda^{ij} (q)

33  virtual const Eigen::VectorXd dtau\_dq(P& z) = 0;

34  virtual const Eigen::VectorXd dtau\_dp(P& z) = 0;

35

36  // phi = 0.5 \* log | Lambda (q) | + V(q)

37  virtual const Eigen::VectorXd dphi\_dq(P& z) = 0;

38

39  virtual void sample\_p(P& z, BaseRNG& rng) = 0;

40

41  virtual void init(P& z) { this->update(z); }

42

43  virtual void update(P& z) {

44

45  try {

46  stan::model::gradient(model\_, z.q, z.V, z.g, err\_stream\_);

47  z.V \*= -1;

48  } catch (const std::exception& e) {

49  this->write\_error\_msg\_(err\_stream\_, e);

50  z.V = std::numeric\_limits<double>::infinity();

51  }

52

53  z.g \*= -1;

54

55  }

56

57  protected:

58

59  M& model\_;

60

61  std::ostream\* err\_stream\_;

62

63  void write\_error\_msg\_(std::ostream\* error\_msgs,

64  const std::exception& e) {

65

66  if (!error\_msgs) return;

67

68  \*error\_msgs << std::endl

69  << "Informational Message: The current Metropolis proposal is about to be "

70  << "rejected because of the following issue:"

71  << std::endl

72  << e.what() << std::endl

73  << "If this warning occurs sporadically, such as for highly constrained "

74  << "variable types like covariance matrices, then the sampler is fine,"

75  << std::endl

76  << "but if this warning occurs often then your model may be either severely "

77  << "ill-conditioned or misspecified."

78  << std::endl;

79

80  }

81

82  };

83

84  } // mcmc

85

86 } // stan

87

88

89 #endif

stan::mcmc::base\_hamiltonian::sample\_p

virtual void sample\_p(P &z, BaseRNG &rng)=0

stan::mcmc::base\_hamiltonian::update

virtual void update(P &z)

**Definition:** base\_hamiltonian.hpp:43

stan::mcmc::base\_hamiltonian::dphi\_dq

virtual const Eigen::VectorXd dphi\_dq(P &z)=0

stan::mcmc::base\_hamiltonian::model\_

M & model\_

**Definition:** base\_hamiltonian.hpp:59

stan::mcmc::base\_hamiltonian::T

virtual double T(P &z)=0

stan::mcmc::base\_hamiltonian::~base\_hamiltonian

~base\_hamiltonian()

**Definition:** base\_hamiltonian.hpp:22

stan::model::gradient

void gradient(const M &model, const Eigen::Matrix< double, Eigen::Dynamic, 1 > &x, double &f, Eigen::Matrix< double, Eigen::Dynamic, 1 > &grad\_f, std::ostream \*msgs=0)

**Definition:** util.hpp:405

stan::mcmc::base\_hamiltonian::dtau\_dp

virtual const Eigen::VectorXd dtau\_dp(P &z)=0

stan::mcmc::base\_hamiltonian::V

double V(P &z)

**Definition:** base\_hamiltonian.hpp:25

stan::mcmc::base\_hamiltonian::write\_error\_msg\_

void write\_error\_msg\_(std::ostream \*error\_msgs, const std::exception &e)

**Definition:** base\_hamiltonian.hpp:63

stan::mcmc::base\_hamiltonian::tau

virtual double tau(P &z)=0

util.hpp

stan::mcmc::base\_hamiltonian::base\_hamiltonian

base\_hamiltonian(M &m, std::ostream \*e)

**Definition:** base\_hamiltonian.hpp:21

stan::mcmc::base\_hamiltonian::init

virtual void init(P &z)

**Definition:** base\_hamiltonian.hpp:41

Eigen.hpp

stan::mcmc::base\_hamiltonian::phi

virtual double phi(P &z)=0

stan::mcmc::base\_hamiltonian

**Definition:** base\_hamiltonian.hpp:17

stan::mcmc::base\_hamiltonian::dtau\_dq

virtual const Eigen::VectorXd dtau\_dq(P &z)=0

stan::mcmc::base\_hamiltonian::err\_stream\_

std::ostream \* err\_stream\_

**Definition:** base\_hamiltonian.hpp:61

stan::math::e

double e()

Return the base of the natural logarithm.

**Definition:** constants.hpp:86

stan::mcmc::base\_hamiltonian::H

double H(P &z)

**Definition:** base\_hamiltonian.hpp:30


---

[ Stan Home Page ]

*© 2011–2014,
Stan Development Team.*
